# Intramedullary Metastases to Conus Medullaris: A Review of the Literature with a Case Illustration

**DOI:** 10.3390/brainsci14080761

**Published:** 2024-07-29

**Authors:** Serdar Kaya, Fred C. Lam, Mary Ann Stevenson, Rouzbeh Motiei-Langroudi, Ekkehard M. Kasper

**Affiliations:** 1 Department of Neurosurgery, Brigham and Women’s Hospital, Boston, MA 02115, USA; 2 Department of Neurosurgery, St. Elizabeth’s Medical Center, Boston, MA 02135, USA; 3 Department of Radiation Oncology, Beth Israel Deaconess Medical Center, Boston, MA 02215, USA; 4 Department of Neurosurgery, University of Kentucky, Lexington, KY 40506, USA; 5 Faculty of Health Sciences, McMaster University, Hamilton, ON L8S 4L8, Canada

**Keywords:** metastasis, conus medullaris, intramedullary, surgery, survival

## Abstract

**Introduction:** Intramedullary metastases to the conus medullaris spinalis (IMCM) pose a rare problem in neurosurgical oncology and are usually encountered as a complicated clinical scenario in the setting of advanced systemic malignancy with poor overall survival. Despite the progress in interdisciplinary oncological care, their management remains complicated. **Research Question:** We performed a PRISMA-guided literature search to achieve a pooled analysis of all previously reported IMCM cases that contained detailed clinical data on this problem to investigate the currently employed management options and respective outcomes. We obtained a clinical vignette and performed a comprehensive narrative review of IMCM management. **Materials and Methods:** The PubMed/MEDLINE/Google Scholar, Cochrane and Embase databases were systematically searched according to the Preferred Reporting Items for Systematic Reviews and Meta-Analyses (PRISMA) guidelines. All relevant publications retrieved were subjected to full-text analysis in detail and pertinent information was extracted. **Results:** The most common systemic primary tumor site as the origin of IMCM was the lung, followed by the breast. Overall, the pooled median survival was 6 months (range 0.5–36 months). Patients who received both surgery and radiation therapy had the longest overall survival (OS) (mean 9.9 months) and those who received no oncological treatment (neither surgery nor adjuvant therapy) had the shortest OS (mean 3.6 months). In cases where surgical resection was performed as part of the treatment plan for metastases, those with partial tumor resection had a more favorable neurological outcome than patients who underwent aggressive gross total resection. **Conclusions:** Based on the results of our analysis, we find that diligent microsurgical resection (subtotal or total) followed by radiation therapy appears as an effective and suitable treatment in select patients with IMCM. When surgery is not feasible as part of the treatment algorithm, radiation therapy alone (conventional or radiosurgery) also appears to be a suitable treatment option that confers a benefit to the patient.

## 1. Introduction

Intramedullary spinal cord metastases (ISCM) are rare and mostly late manifestations of systemic malignancy. Such lesions are seen in as many as 0.1–0.4% of all cancer patients [1]. Unknown to most oncological providers, ISCM constitute about 4.2 to 8.5% of all metastatic deposits to the central nervous system (CNS) and nearly 3.5% of all spinal metastases. However, the ISCM incidence in select autopsy series was reported in only 0.9 to 3.9% of the identified metastatic sites [2,3]. Albeit rare, the relevant debilitating outcomes make this problem noteworthy, and we have identified a knowledge gap in the treatment provider community regarding the best treatment options. However, advances in cancer therapeutics and improvements in cancer-specific survival will increase the number of ISCM cases as other metastases, and there will be more publications on this rare topic [4]. We wish to contribute to the advancement of knowledge regarding this complicated scenario with this study and encourage the further investigation of the best treatment algorithms by creating multicenter registries for this condition.

The overall survival of patients with ISCM is rather poor and ranges from 4 to 17 months [5], which likely reflects the advanced disease status. Multiple treatment modalities, including chemotherapy, radiotherapy, radiosurgery and surgery, have been advocated for in this setting, and surgery and/or radiotherapy are considered to exert a beneficial impact on overall survival [5,6,7]. Nevertheless, given the rather low incidence of this clinical problem, no sizable cohort study has been performed to date, indicating a gap in high-quality data in the literature, especially with respect to histopathologically specific recommendations. This has also prevented the establishment of an acknowledged treatment strategy.

IMCM are considerably less frequent than intramedullary spinal cord metastases (ISCM), but a specific epidemiological description of their prevalence is lacking, likely due to the very low number of observed cases. Most IMCM encounters have been published as simple case reports or included in small ISCM series. We thus wish to address the existing knowledge gap with this investigation. Thus, we sought to identify and review all IMCM cases reported to date in the English-language literature. This was undertaken in order to elucidate the best possible management strategy for the treatment for this rare entity.

## 2. Materials and Methods

### 2.1. Literature Search Strategy

**Figure 1:**
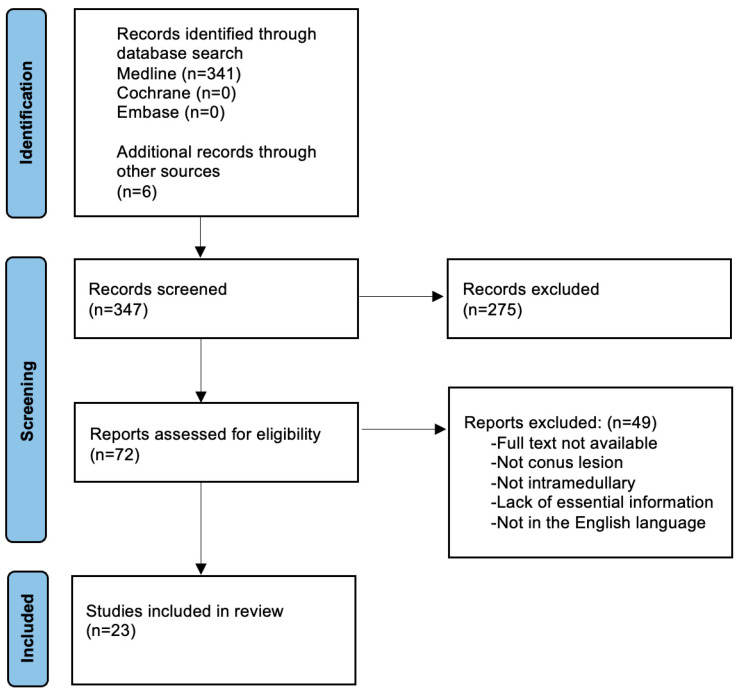
PRISMA 2020 flow diagram depicting the process of searching for, including and excluding studies on intramedullary conus medullaris metastases.

We reviewed the PubMed/MELINE, Google Scholar, Embase and Cochrane databases for any articles on the topic. To capture valid reports from the post-MRI era, we restricted our search to those published after 1990 and until November 2023. Three search terms were employed in a Boolean algorithm: spinal cord, metastasis and intramedullary. The search strategy was carried out with the help of a qualified librarian and is detailed in Figure 1. The search followed the Preferred Reporting Items for Systematic Reviews and Meta-Analyses (PRISMA) 2020 guidelines for the reporting of systematic literature research (Figure 1) [8]. This systematic review protocol was designed a priori and is registered in PROSPERO (CRD42021288965).

The criteria for the inclusion of relevant articles in the review were as follows: (1) published in academic, peer-reviewed journals as case reports, case cohort/series, clinical trials and reviews if these contained granular patient data; (2) English language; (3) adults only (>18 years old) and human studies; (4) a confirmed pathological diagnosis of a primary neoplasm or new histopathology from surgery; (5) the availability of essential information, such as the presenting signs and symptoms, imaging data, treatments delivered, neurological status before and after the treatment and follow-up. Studies not meeting these inclusion criteria were excluded.

### 2.2. Data Extraction

The titles and abstracts were screened by 2 reviewers independently (R.M., S.K.). In the event of any discrepancy during screening, articles were automatically included for further evaluation, to ensure that all relevant articles were involved. Abstracts that met the selection criteria mentioned above were then retrieved in full-text form. Two reviewers independently conducted data extraction. Information obtained from the qualifying studies included the number of patients and their gender, age, signs and symptoms, neurological status before and after treatment, treatment, histopathology, the presence of metastases in other organs, the interval from the diagnosis of the primary tumor to the diagnosis of IMCM and survival. Discrepancies observed in the full text and data abstraction stages were resolved by consensus between reviewers, or the senior author (EMK) was consulted as the third reviewer. This information was then used to construct a combined evidence table (Table 1).

### 2.3. Case Illustration

A patient with a symptomatic conus medullaris metastasis presented to our institution. She was a 51-year-old non-smoking female with a known history of metastatic renal cell carcinoma (RCC stage 4, T3N2M1). The only other noteworthy aspects of her medical history were a benign breast adenoma, cholecystectomy, hyperlipidemia and depression. The primary RCC was diagnosed and treated by a left radical nephrectomy and retroperitoneal lymph node dissection, followed by two cycles of high-dose interleukin 2 (IL-2). Four months later, she also underwent video-assisted complete left lingual wedge resection because of a newly identified pulmonary metastasis. Thirteen months after her primary cancer diagnosis, she was diagnosed with an isolated lesion to the conus (IMCM), which was identified after a work-up for radicular low back pain. Her lower extremity strength and sensation were intact and she denied incontinence. At the time of evaluation, this was the only active systemic disease site and the decision was made to treat her with fractionated stereotactic radiation therapy (2500 cGy total dose: first course 1500 cGy (500 cGy ×3), cone down 1000 cGy (500 cGy ×2), open field for both, hand calculation, no 3D graphic plan) and bevacizumab (10 mg/kg for a total of 15 cycles) and axitinib (started 5 mg twice daily and continued with 5 mg daily) under dexamethasone coverage. Post-treatment MR imaging remained initially stable. Her conus tumor, however, then progressed clinically further with bilateral lower extremity weakness, and, 49 months after her IMCM diagnosis, she was treated with a course of bevacizumab.

**Figure 2:**
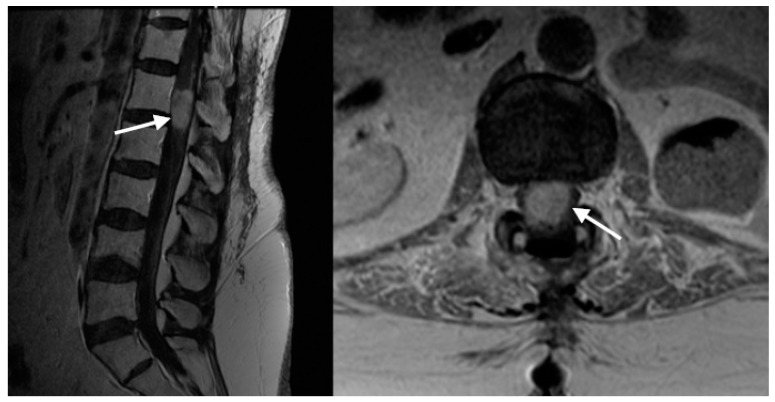
Sagittal and axial contrast-enhanced T1W MRI images show an enhancing lesion, compatible with conus medullaris metastasis (white arrows).

Two months later, she underwent ventriculoperitoneal shunt placement due to the development of symptomatic hydrocephalus. CSF cytology did not show malignant cells at that time. Over time, her paraparesis progressed further to 1/5 strength in her right and 2/5 in her left lower limb throughout, as well as complete bladder and bowel incontinence. Additionally, she was suffering from intractable lower back pain and radicular leg pain despite using strong pain medication (fentanyl, gabapentin, haloperidol, lidocaine patch and oxycodone). Subsequent imaging studies showed that the size of the previously treated IMCM had increased to 35 mm × 22 mm × 18 mm and the lesion displayed profound peritumoral edema, cord expansion and a large thoracic syrinx (Figure 2).

Given the fact that her pain was not fully controlled by palliative measures, she consented to surgical intervention by another neurosurgical provider and she underwent a T12-L2 laminectomy and subtotal tumor resection (approximately 50% debulking). Postoperatively, her paraparesis transiently improved in all muscle groups in the bilateral lower limbs. However, her perioperative course was complicated by recurrent urinary tract infections and sepsis, pulmonary embolism and wound complications (surgical site infection), which were all treated appropriately. After extensive discussions within the treating medical oncology team and with the patient and her family, she then decided to pursue palliative care only emphasizing QOL with prolonged physical therapy and pain management. She finally passed away about 55 months after her initial IMCM diagnosis.

## 3. Results of Our Literature Search

### 3.1. Studies and Patient Data

Twenty-three studies comprising detailed reports on 50 individual neurooncological cases were included in the final analysis for the current review. Overall, the data retrieved from seven studies revealed an unclear risk of bias. The clinical information of all patients is summarized in Table 1. We then pooled the data set for a group analysis, as presented below.

### 3.2. Epidemiology

In the entire pooled cohort of reported IMCM patients (with varying underlying cancer types), the mean age at the diagnosis of the problem was 57.2 ± 12.4 years (range 33 to 82 years). There were 26 males and 24 females (M/F ratio 1.08) in this cohort.

The most common source of IMCM was primarily the lung (20 cases or 40%), followed by the breast (10 cases or 20%). This was expected based on published primary tumor frequencies in an unselected community in the USA. The mean interval from the diagnosis of the primary tumor to the time of the diagnosis of IMCM (primary to metastasis interval) was 30.5 months (range 0 to 144 months). The interval between the primary cancer diagnosis and IMCM was significantly shorter in patients with lung cancer (12.1 months) than patients with breast cancer and other primaries (48.6 and 40.8 months, respectively), (*p* = 0.009). Of the entire cohort, 25 patients (54.3%) had been diagnosed with other synchronous active systemic metastases at the time of their IMCM diagnosis. The most frequent site of synchronous metastasis was the brain, followed by the liver and lung. These epidemiological data are summarized in Table 2.

### 3.3. Signs and Symptoms

The IMCM symptoms varied widely, with the most common clinical symptom reported as sensory changes, followed by bowel/sphincter dysfunction, pain and lower extremity weakness (manifested as either mono- or paraparesis) (Table 1). Sexual dysfunction was not investigated or separately reported in the articles involved in this study.

### 3.4. Treatment

Forty-two of the patients in this cohort (84%) underwent some form of open microsurgery for the resection or debulking of the IMCM lesion. Among these operated patients, surgeons achieved a microscopic gross total resection in only 8 of the 42 cases. Twenty-three patients went on to receive further adjuvant care and received postoperative radiation therapy as an adjuvant treatment. No neo-adjuvant radiation therapy has been reported to date. Three patients did not undergo microsurgery or radiation and were treated medically, and five patients were treated with radiation therapy alone.

### 3.5. Outcomes and Survival

The neurological status improved in 60.4% of the pooled patient cohort, and this status remained stable in as many as 27% of the patients after the completion of treatment (either surgery or radiation therapy). Sex did not influence the post-treatment neurological outcome (*p* = 0.89). The primary tumor pathology as the source of the metastatic deposit and the presence of further systemic metastases, however, significantly influenced the neurological outcome (*p* = 0.012, <0.001, respectively), with less favorable results in patients harboring lung cancer as the primary source, compared to those with other primary neoplasms. Of note, none of these parameters had an effect on overall survival (*p* = 0.6, *p* = 0.6). Moreover, the neurological improvement was superior in those patients who underwent surgery or radiation (*p* = 0.015 and 0.012, respectively). However, a clear caveat needs to be expressed with regard to these observations, given the low number of available cases for analysis, which rendered the study outcomes prone to selection bias.

In patients who had surgery for the resection of the IMCM, the neurological status before surgery (in terms of muscle weakness) was not different in partially vs. totally resected patients (*p* = 0.75). However, those patients in whom only partial tumor resection was accomplished had a more favorable neurological outcome than patients who lived with totally resected lesions (*p* = 0.07). Overall, patients who underwent both open microsurgery and adjuvant therapy had the most favorable neurological outcomes (83.3% improved and 12.5% remained stable over the observation period), followed by those who had surgery alone (35.2% and 58.8% improvement and stable neurological outcome, respectively). This compares favorably to those rates obtained in patients who underwent radiation therapy without prior surgery (50% and 50% improvement and worsened neurological outcome, respectively). These were managed without any therapeutic active intervention and treated conservatively only for palliative symptom control (e.g., pain medication and steroids); they had no benefit from the intervention and exhibited the worst neurological outcomes (100% worsening) (*p* = 0.002).

Overall, the median survival of the mixed pooled cohort was 6 months (range 1–36 months), which may not be a relevant measure given the various histopathologies represented in the study cohort.

There was an (albeit non-statically significant) positive correlation between the primary to metastasis interval and survival (r = 0.2, *p* = 0.29). Neither the primary site of IMCM nor the presence of other systemic metastases determined the overall survival (*p* = 0.6 and 0.32, respectively). Of note, different age or sex groups had similar survival rates (*p* = 0.88, 0.97, respectively).

The mean survival was improved with all provided treatment options (surgery and radiation therapy, surgery alone and radiation therapy alone vs. palliative care = 9.9 months, 6.6 months, 6.7 months, respectively, vs. 3.6 months), although the between-group difference was not statistically significant (*p* = 0.35). Survival appeared slightly longer in patients in whom gross total resection had been achieved vs. those patients in whom the resection remained subtotal/partial, although this difference did not reach statistical significance either (*p* = 0.90). The survival of patients who worsened clinically postoperatively or who had an unchanged neurological status after treatment was also shorter (1.6 months) than the mean survival of the entire cohort.

## 4. Discussion

ISCM are quite rare and accompanied in most patients by a rapid decline in neurological status. As the use of MR imaging in cancer diagnostics has become more popular, more cases of ISCM and its unique subgroup of IMCM are being discovered. Given the paucity of available data, it remains uncertain how to clinically manage this scenario.

In this review, we investigated whether cases with IMCM have presented with conus medullaris syndrome (CMS) findings. CMS is usually defined as a spinal cord injury at the vertebral level of Th12-L2, with significant back pain, saddle anesthesia, bladder and/or bowel disturbances or anal problems, sexual problems and little to no motor impairment in the lower limbs [30]. The present cases were either not investigated for sexual dysfunction or this was not a presenting symptom. Overall, 72% of the cases included in our cohort had some degree of paresis. With these findings, it is not possible to state that IMCM would likely or most often present with CMS.

The survival of patients diagnosed with IMCM is rather poor. Patients usually suffer from a profound neurological status decline and a significant loss of quality of life [6]. In our analysis, the mean age upon presentation was 56 years, which is consistent with previous reports [3,5,9,14]. However, patients with metastases from a primary breast cancer were younger than those with lung cancer. Lung cancer was the most frequent primary source, followed by breast cancer, which reflects the epidemiological landscape observed in an unselected patient cohort.

In our pooled cohort analysis, the results demonstrated no significant differences in IMCM in patients with lung cancer, breast cancer and the remaining pathology subgroups in terms of survival or symptomatology. However, those with lung cancer as the primary source had less favorable neurological outcomes over time. Hsu et al. reported that patients with breast cancer as the primary source of ISCM had longer overall survival of 13 months, compared with 3 months for patients with primary tumors of the lung [10]; in our series, however, the median survival of the two groups was not significantly different. This may be due to the clinically different disease courses and outcomes in IMCM compared to ISCM. Of note, the period from the diagnosis of the primary tumor until the diagnosis of IMCM was 48.6 months in breast cancer patients, four times longer than the period seen in lung cancer patients.

Four patients in our pooled cohort (9.3%) had no prior history of systemic carcinoma. Presentation with intramedullary metastasis without a known primary source has been reported in about 25% of ISCM cases, but the respective data set is lacking for IMCM [6]. Such differences in numbers may be due to the different symptomatology, the natural course and the specific aspects of the tumor location in IMCM and ISCM. For instance, 48.8% of IMCM patients suffered sphincter dysfunction at presentation, which was notably different from patients with ISCM, who predominantly reported pain, weakness or paresthesia [14,31,32].

Goyal et al. stated that the presence of intramedullary metastasis had a greater impact on survival than the systemic metastatic burden [33]. In our review, the survival rates for cases with and without systemic metastases were similar, supporting this notion. However, this discrepancy may be attributable to the low number of available cases for analysis, since it is not in line with the classical observation of cancer survival, which reflects the potential benefits of diagnosing all cancer types early and less advanced, which reduces mortality.

The management strategies for IMCM are not well defined and clinical efforts should focus on preventing the deterioration of neurological symptoms. Different approaches and modalities have been used to treat these complex and difficult-to-manage lesions. Radiotherapy alone has shown some efficacy in arresting or slowing tumor growth and preventing further neurological deficits in some cases, but the absence of pathological data and the rapid evolution and progress of neurological deficits before effect onset limit its application [30,34]. Published reports have demonstrated that surgery extends survival and also tends to improve the neurological status [5,9,10].

Our study shows that surgical resection followed by adjuvant therapy (with specific modalities chosen based on the tumor source and pathology) culminates in the best results in terms of both survival and post-treatment neurological outcomes. Either surgical resection or radiation alone was associated with better neurological outcomes, even though these interventions did not increase survival. We believe that both should be considered in patients with IMCM in cases when there are no surgical and medical hazards.

Total microsurgical resection, on the other hand, did not confer a survival benefit, i.e., significantly prolonging the median survival when compared to patients undergoing subtotal or partial resection and radical resection. It was in fact associated with worse neurological outcomes (and diminished quality of life). Therefore, based on the current analysis of reported cases from the literature, we recommend performing diligent microsurgical exploration under intraOP monitoring conditions and possibly accepting a subtotal resection instead of pursuing an aggressive attempt at gross total resection in cases when surgery seems technically challenging.

During our data extraction process, the abstracts of articles that had not been published in the full text in the English language were further screened so as not to miss any other large series of ISCM, but we encountered only two case reports of ISCM in Japanese. These articles were excluded from our detailed review [1,35]. Nevertheless, we are aware of the fact that this analysis is significantly limited by the low total number of identified patients with granular and complete data sets, which limits the power of the conclusions drawn. This is a limitation that needs to be addressed by creating a multisite registry of such rare cases or by coordinating larger multicenter studies to overcome the constraints posed by the very low incidence of IMCM in surgical practice.

Another limitation of the study is the fact that survival is most frequently affected by synchronous metastasis to other systemic sites. In this domain, the results did not reach significance, although they demonstrated worse survival outcomes in patients with other systemic metastases, with a higher number of such metastases; therefore, the calculation of the overall survival is less meaningful, since the uncontrolled systemic disease status may confound the observations made.

One additional point to be stated as a limitation is that all reviewed papers had used external beam radiation therapy, and data regarding more recent modalities, including stereotactic radiosurgery, lack sufficient numbers. The use of stereotactic radiosurgery is on the rise and has shown efficacy in some ISCM [34], especially in tumor pathologies (e.g., RCC) that are considered rather resistant to conventional radiation therapy. Given the shorter course of treatment with radiosurgery, it remains an attractive option in terminal cancer care—as it allows the healthcare provider to keep the affected patients out of the hospital as much as possible and to provide effective care with a limited time commitment on an outpatient basis. Its benefit and efficacy in IMCM should hence be further evaluated in future studies.

Based on the results of this meta-analysis, we propose a treatment algorithm that incorporates an attempt at an initial microsurgical tumor resection (partial or subtotal) followed by adjuvant radiation in the form of external beam radiation or stereotactic radiosurgery as the treatment of choice in IMCM. Chemotherapy can be added as well if the respective primary pathology permits or warrants it. In the literature, chemotherapy has been used in conjunction with radiotherapy or surgery for the treatment of ISCM but is rarely used as a monotherapy [14]. Chemotherapy may provide additional benefits in patients who have not been heavily pretreated with neo-adjuvant chemotherapy or if there are extensive systemic metastases, particularly cases with chemosensitive tumors, such as small-cell lung cancers and hematological malignancies. Nevertheless, studies on the efficacy of chemotherapy in ISCM are limited and show no effect on overall survival [18,36]. Thus, we did not include chemotherapy as one of the main treatment modalities for IMCM.

Our analysis does not support aggressive attempts at total resection as it results in worse neurological outcomes and does not translate into the prolongation of survival. In patients in whom not all treatment options are available or feasible (due to surgical risks, patient preferences, anticoagulant drug use, poor medical condition, etc.), radiation alone is the most suitable option as it is accompanied by a lower cost and patient risk but with comparable overall survival when compared with surgery alone. Of note, conservative treatments (including physical therapy, pain medications, steroids, etc.) should be added to the treatment protocol, but the sole use of conservative management is not effective other than for symptom relief in these cases.

## 5. Conclusions

Based on the results provided in detail above, it is reasonable to choose surgery and radiation therapy for the treatment of IMCM over palliative treatment, as it provides improved neurological outcomes and hence enhanced quality of life, albeit not providing evidence of prolonged overall survival.

## Figures and Tables

**Table 1:** Combined evidence table.

Study	Gender	Age	Signs and Symptoms	Primary Tumor Site (Histology)	Other Metastases	Time from Primary to IMCM (Months)	Surgery	Radiation	Follow-Up (Months)	Symptoms Postop
Gasser et al. [3]	M	47	Paraparesis	Epithelioid sarcoma	N/A	N/A	Y	N	24	Unchanged
	M	58	Paraparesis	Adenocarcinoma	N/A	N/A	Y, total	N	7.8	Unchanged
	M	43	Paraparesis	Poorly differentiated Ca	N/A	N/A	Y	N	13.5	Unchanged
	M	69	Paraparesis	Poorly differentiated Ca	N/A	N/A	Y	N	3.2	Unchanged
Payer et al. [4]	M	77	Dysesthesia	Lung, non-small cell Ca	N	0	Y	N	3	Unchanged
	F	56	Dysesthesia, paresis	Breast	Lung, brain	28	Y	N	2	Unchanged
	F	54	Hypoesthesia, pain	Ependymoma Grade III	N	41	Y, total	N	N/A	Unchanged
	M	54	Hypoesthesia, pain	Lung, adenocarcinoma	Brain	46	Y	N	N/A	Unchanged
Dam-Hieu et al. [9]	M	50	Pain, bladder dysfunction	Lung, small cell Ca	N	11	Y	Y	6	Improved
	F	39	Paresthesia, bowel, and bladder dysfunction	Lung, small cell Ca	N	12	N	N	4	Worsened
	M	79	Paraparesis, bowel and bladder dysfunction	Colon	Liver	24	Y	Y	5	Improved
	M	66	Cauda equine syndrome	Lung, adenocarcinoma	Liver, brain	12	N	N	1	Worsened
	M	57	Paraparesis	Lung, adenocarcinoma	N	3	Y	Y	4	Worsened
	M	49	Paraparesis, bowel and bladder dysfunction, pain	Lung	Orbit	18	Y	Y	2	Improved
	F	63	Paraparesis, paresthesia, bladder dysfunction	Breast	N	3	Y	Y	24, alive	Improved
	M	55	Cauda equine syndrome (tumor located also on cauda)	Lung, epidermoid	N	24	Y	Y	11	Improved
	F	68	Paresthesia, gait difficulty	Lung, small cell Ca	N	24	Y	Y	15, alive	Improved
	F	35	Asymptomatic	Breast	Bone	48	N	N	6, alive	N/A
	F	65	Paraparesis, leg pain	Lung, small cell Ca	N	20	Y	Y	4, alive	Improved
Hsu et al. [10]	F	45	Paraparesis, bowel and bladder dysfunction, pain	Breast, infiltrating ductal Ca	Lung, liver, brain	98	Y, total	N	12, alive	Improved
Choi et al. [11]	F	45	Paresis, paresthesia	Breast, infiltrating ductal Ca	Brain, C6	96	Y	N	2	Unchanged
Isla et al. [12]	F	54	Paraparesis, bowel and bladder dysfunction	Breast	Lung, liver, brain	12	Y	Y	5	Improved
Hrabalek et al. [13]	F	48	Paraparesis, paresthesia, bladder dysfunction	Breast, infiltrating ductal Ca	Breast, lymph node	84	Y	Y	4	Improved
Sung et al. [14]	M	63	Paresis, paresthesia	Lung	Liver, brain, bone	1	N	Y	4	Worsened
	F	51	Paresis, pain	Lung	Brain	3	Y	N	2	Worsened
	M	82	Pain	Prostate	N	42	Y	Y	8	Improved
	F	54	Paresis, paresthesia, bowel and bladder dysfunction	Breast	C2, C5	69	N	Y	3	Worsened
Ramakonar et al. [15]	F	44	Paresis, pain, paresthesia	Periurethral adenocarcinoma	Brain	48	Y, total	Y	5, alive	Improved
Wu et al. [16]	M	74	Paresis, pain, hypoesthesia, bowel, and bladder dysfunction	Prostate adenocarcinoma	Bone	N/A	Y, total	N	6, alive	Improved
Guppy et al. [17]	M	54	Paresis, pain, bladder dysfunction, paresthesia	Lung, infiltrating differentiated adenocarcinoma	Liver, brain	N/A	Y	N	2	Improved
Sutter et al. [18]	F	40	Paraparesis, bladder dysfunction	Breast	Diffuse	N/A	Y, total	Y	19	Improved
	M	54	Ataxia, bladder dysfunction, paresis	Lung, large cell Ca	N	N/A	Y	N	6, alive	Improved
	M	45	Paraparesis, bowel and bladder dysfunction, paresthesia	Lung, oat cell Ca	N	N/A	Y	N	8	Improved
Wostrack et al. [19]	F	73	Paresis, bladder dysfunction, dysesthesia	Ovarian Ca	Lung	28	Y, total	Y	7.5	Unchanged
Yang et al. [20]	M	75	Paresis, pain, numbness	Rectum	Brain	48	N	Y	4	Improved
Jeon et al. [21]	F	44	Paresis, pain, bowel dysfunction	Thyroid, papillary Ca	Lung, brain	144	Y	Y	15, alive	Improved
Khoshnevisan et al. [22]	F	61	Paresis, pain, bladder dysfunction	Endometrial Ca	N	15	Y	Y	18, alive	Improved
Fakih et al. [23]	F	68	Paresis	Renal cell Ca	N	2	N	Y	16	Improved
Kaya et al. [24]	M	43	Paresis, pain, bowel and bladder dysfunction	Renal cell Ca	N	12	Y, total	N	6	Improved
Mavani et al. [25]	M	46	Paresis, bowel and bladder dysfunction, sensory impairment	Lung, poorly differentiated carcinoma	N	0	Y, partial	Y	1, alive	Improved
Li et al. [26]	F	33	Paresis, bladder dysfunction, back pain	Bronchogenic carcinoid	Cervical cord, brain	48	Y, partial	Y	Alive	Improved
Marchesini et al. [27]	M	54	Paresis, pain, numbness, bladder dysfunction	Adenoid cystic carcinoma	Brain	121	Y	N	3	Unchanged
Gazzeri et al. [28]	F	67	Paresis, pain, numbness, bladder dysfunction	Kidney	N	8	Y	Y	6	Improved
	M	44	Pain, bladder dysfunction	Lung	Yes	14	Y	Y	4	Improved
	F	70	Pain, numbness, bladder dysfunction	Lung	N	11	Y	Y	7	Improved
	M	66	Pain, numbness	Lung	N	0	Y	Y	17	Unchanged
	F	72	Paresis, numbness	Breast	N	0	Y	Y	5	Unchanged
	M	65	Paresis, bladder dysfunction	Melanoma	Yes	18	Y	N	11	Improved
	M	76	Paresis, numbness	Lung	Yes	8	Y	Y	36	Improved
Kalimuthu et al. [29]	M	65	Backpain	Renal Cell carcinoma	N	6	N	Y	N/A	N/A

**Table 2:** Epidemiological data of cases.

Primary Source	Number of Cases	Mean Age, Years	Median Survival, Months	Time from Primary to IMCM (Months)	Number (%) of Patients with Other Metastasis
Lung	20	57.45	4.0	12.1	8 (40%)
Breast	10	51.2	5.0	48.6	8 (80%)
Other	20	59.9	7.8	38.8	9 (56.2%)
Total	50	56.3	6.0	33.2	25 (54.3%)

## Abbreviations and Acronyms

IMCM	Intramedullary metastases to the conus medullaris
ISCM	Intramedullary spinal cord metastases
CNS	Central nervous system
PRISMA	Preferred Reporting Items for Systematic Reviews and Meta-Analyses
